# Emergence and Epidemiology of Bovine Babesiosis Due to *Babesia divergens* on a Northern German Beef Production Farm

**DOI:** 10.3389/fvets.2020.00649

**Published:** 2020-09-15

**Authors:** Andrea Springer, Martin Höltershinken, Fabienne Lienhart, Sandra Ermel, Jürgen Rehage, Kirsten Hülskötter, Annika Lehmbecker, Peter Wohlsein, Dieter Barutzki, Christine Gietl, Wolfgang Baumgärtner, Martina Hoedemaker, Christina Strube

**Affiliations:** ^1^Institute for Parasitology, Centre for Infection Medicine, University of Veterinary Medicine Hannover, Hanover, Germany; ^2^Clinic for Cattle, University of Veterinary Medicine Hannover, Hannover, Germany; ^3^Department of Pathology, University of Veterinary Medicine Hannover, Hanover, Germany; ^4^Veterinary Laboratory Freiburg GmbH, Freiburg, Germany; ^5^MEGACOR Diagnostik GmbH, Hörbranz, Austria

**Keywords:** tick-borne diseases, vector-borne diseases, cattle, haemoglobinuria, zoonosis, *Babesia divergens*, *Babesia microti*, *Babesia venatorum*

## Abstract

*Babesia divergens*, transmitted by the tick *Ixodes ricinus*, is the most common cause of bovine babesiosis in northern Europe and plays a role as a zoonotic pathogen. However, several studies have indicated a decline of *B. divergens* prevalence in Europe during the last decades. Here, we investigate the epidemiology of bovine babesiosis on a beef production farm in northern Germany, which had not been affected by babesiosis until an initial outbreak in 2018. In June 2018, 21 adult cattle died, showing classical symptoms of babesiosis. *Babesia divergens* merozoites were detected in blood smears of clinically affected animals and the species was confirmed by PCR and sequencing of a part of the 18S rRNA gene. In 2018, screening of the farm's entire stock by PCR revealed that *Babesia*-positive animals were present in only one of five herds grazing on different pastures. In the following year, further babesiosis cases occurred in multiple herds. In March 2020, 95 cattle were tested for anti-*B. divergens* antibodies and 36 of them (37.89%) had positive titres. To investigate the local *Babesia* prevalence in ticks, 1,430 questing *I. ricinus* ticks (555 larvae, 648 nymphs, 227 adults) were collected on the farm's pastures and subjected to PCR for *Babesia* detection. *Babesia divergens* DNA could not be detected, but *Babesia microti* showed an overall prevalence of 0.49% (7/1,430; 0.88% [2/227] of adult ticks, 0.77% [5/648] of nymphs, 0.00% [0/555] of larvae). *Babesia venatorum* was detected in 0.42% (6/1,430) of ticks (0.44% [1/227] of adult ticks, 0.77% [5/648] of nymphs, 0.00% [0/555] of larvae) and *B. capreoli* in 0.07% (1/1,430) of ticks (0.00% [0/227] of adult ticks, 0.15% [1/648] of nymphs, 0.00% [0/555] of larvae). Despite the fact that no *B. divergens*-positive ticks were found, the collected data suggest a geographical spread of the pathogen on the farm. Bovine babesiosis remains a disease of veterinary importance in Europe and may cause considerable economic losses when (re-)emerging in non-endemic areas, especially as awareness for the disease among veterinarians and farmers declines.

## Introduction

Protozoan parasites of the order Piroplasmida are the most important blood parasites of domestic animals. They are transmitted by ticks, representing the definitive hosts, to the vertebrate intermediate host during the tick's blood meal. In the vertebrate host, they replicate in erythrocytes, causing potentially fatal haemolytic anemia. In cattle, *Babesia bigemina* and *Babesia bovis* are of worldwide economic importance (1), whereas in European countries located north of the Alps, *Babesia divergens* is the main cause of bovine babesiosis (2). Furthermore, *B. divergens*—amongst other *Babesia* spp.—may cause potentially fatal disease in humans, primarily in splenectomized or immunologically compromised people, but also in immunocompetent individuals (3).

*Babesia divergens* is primarily transmitted by the sheep or castor bean tick *Ixodes ricinus*, which is the dominant tick species throughout Europe, although the Asian lineage of *B. divergens* is probably transmitted by *Ixodes persulcatus* (4). Both species are three-host ticks and usually require 3–6 years to complete their life cycle (5). Each of the three life stages (larva, nymph, adult) takes one blood meal before development to the next stage or, in case of females, oviposition, whereas adult males rarely take a blood meal. Larvae prefer small rodents and birds as hosts, while nymphs and adults prefer larger mammals, including cattle (6). The main habitats of *I. ricinus* are forests, woodlands and shrubs. While well-maintained pastures represent an inferior habitat, hedges and pockets of forest between pastures may sustain *I. ricinus* populations and represent foci of *B. divergens* transmission (7). Transovarial as well as transstadial transmission in ticks facilitates persistence of *B. divergens* in such foci, even in the absence of the vertebrate intermediate host (8).

In cattle, a so-called “inverse age resistance” leads to subclinical or mild infections with induction of immunity in animals under the age of ~9 months (9, 10). Immunity is then maintained by repeated pathogen exposure (8). Thus, clinical cases are rare in endemic regions and usually affect immunologically naïve, recently introduced cattle or animals that had no access to pasture in the first year of life (8). Clinical signs consist of elevated body temperature, anorexia, weakness and haemolytic anemia accompanied by tachycardia and tachypnoea. Mucous membranes may be pale or jaundiced, and haemoglobinuria occurs at the peak of the haemolytic crisis (8, 11). Spontaneous recoveries are rare, thus, case fatality rates are mainly influenced by the speed of diagnosis and treatment (12).

Several studies have indicated a change in *B. divergens* epidemiology in Europe during the last decades. A marked decrease in disease incidence has been reported from Norway (13), Hungary (14), and Ireland (12). A reduction in cattle density, changes in pasturing practices and improvement of pastures have been proposed as causative factors for this decline (12–14). In Ireland, where bovine babesiosis was once a disease with considerable economic impact, incidence has declined considerably since the 1980s, and, furthermore, the annual pattern of disease outbreaks has changed (12). Bovine babesiosis cases usually occur in a bimodal pattern, peaking in spring/early summer (April to June), and again in autumn (August to October), mirroring the activity peaks of *I. ricinus* (8, 15). However, the Irish study reported that recent cases occurred throughout the year, and that disease severity and case fatality had increased as compared to the past, possibly due to lower awareness for the disease resulting in delayed diagnosis and treatment (12).

The most recent available data regarding *B. divergens* in northern Germany date back to the year 1990, when an epidemiological study reported 1.3% *B. divergens*-seropositive cattle (*n* = 766) (16). Since then, the lack of studies indicates that the disease has been of little economic importance in northern Germany. Similar to the other European countries mentioned above, it is possible that disease incidence has been declining in northern Germany during the past three decades. As a downside, declining *B. divergens* prevalence results in reduced herd immunity and the loss of endemic stability (17). Here, we describe the epidemiology of bovine babesiosis with a high fatality rate on an extensively managed beef production farm in northern Germany, which had not been affected by *B. divergens* in the past.

## Materials and Methods

### Farm Characteristics and Outbreak Description

A babesiosis outbreak occurred on an organic beef production farm in northern Germany. In May 2018, the farm's stock included approximately 150 German Red Pied and Angus cattle in five extensively managed suckler herds grazing on different pastures. Most animals had been born and raised on the farm. The farm's pastures bordered and partially extended into a nature reserve characterized by moorland, grassland, and the presence of a lake. The nature reserve also serves as a popular recreational area.

**Figure 1:**
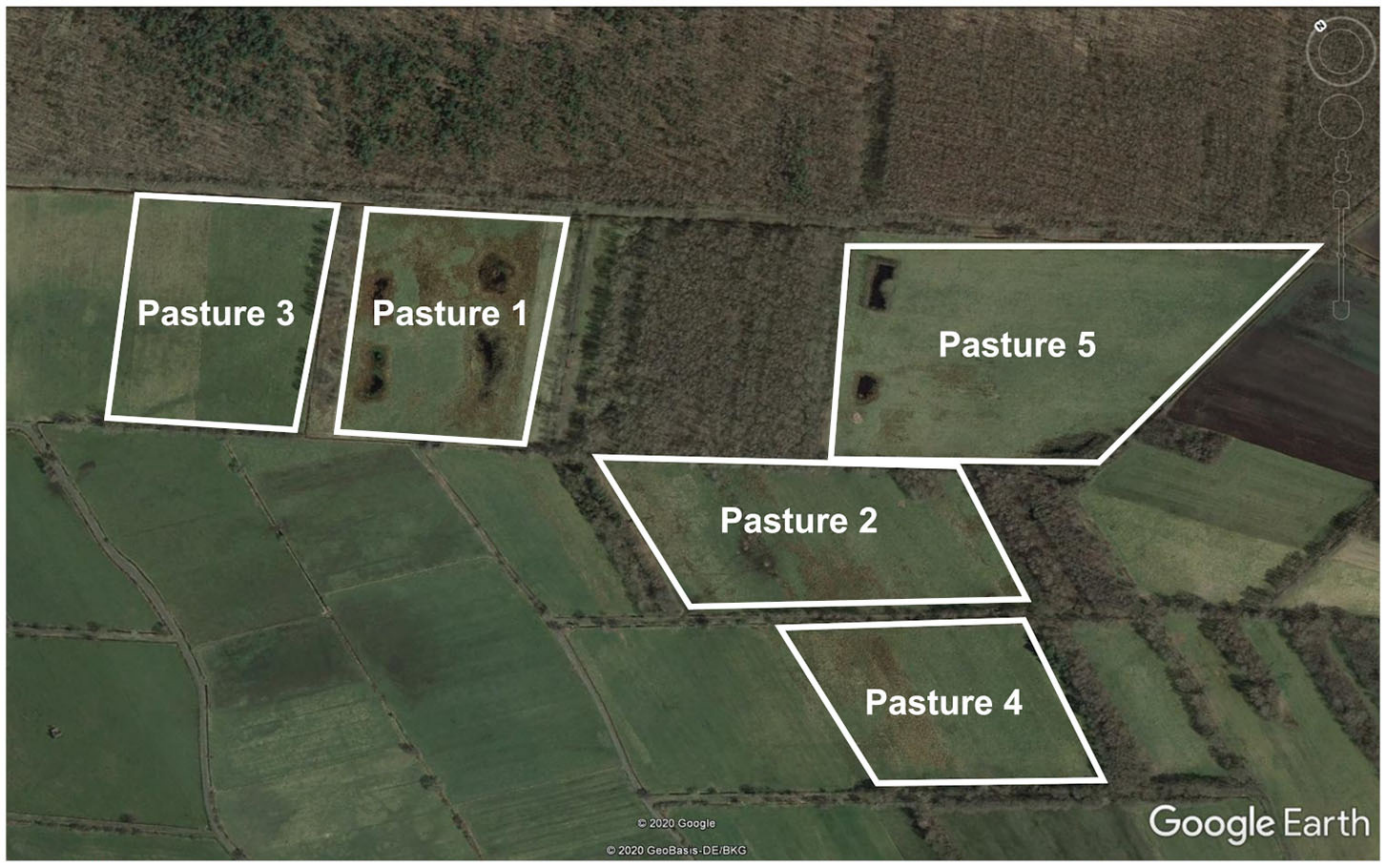
Satellite image of the pastures where babesiosis cases occurred and/or ticks were flagged for assessment of *Babesia* prevalence. Source: Google Earth v. 7.1.8.

The beginning of the outbreak has been briefly summarized previously in an overview on babesiosis diagnosis and management for local veterinarians (18) and the development of an *in situ* hybridization for *post mortem* diagnosis (19). In detail, haemoglobinuria was first noted in one of the farm's herds, consisting of 56 animals, on June 3rd 2018. On May 25th (10 days earlier), the herd had been led onto a pasture which had not been used previously during that year (“pasture 1,” Figure 1). On June 5th, six animals (one bull and five cows) were found dead on the pasture, followed by one further death of a cow on June 6th (Table 1). At this point, poisoning of the natural ponds on pasture 1 was considered as a cause of the fatalities, thus, the herd was moved onto another pasture (“pasture 2,” Figure 1).

**Table 1:** Series of events during a *Babesia divergens* outbreak on a Northern German cattle farm in 2018 and 2019.

**Date**	**Event**
25th May 2018	“Herd 1” moved to “pasture 1”
3rd June 2018	First observation of haemoglobinuria in “herd 1”
5th June 2018	Death of 6 cattle from “herd 1”
6th June 2018	Death of 1 cattle from “herd 1”
7th June 2018	Death of 2 cattle from “herd 1”
	“Herd 1” moved to “pasture 2,” joined by 20 cows from “herd 2” (originally on “pasture 3”)
8th June 2018	Death of 2 cattle from “herd 1” on “pasture 2”
10th June 2018	Death of 1 cattle from “herd 1” on “pasture 2”
14th June 2018	Death of 1 cattle from “herd 1” on “pasture 2”
19th June 2018	Death of 4 cattle on “pasture 2,” originally from “herd 2”
20th June 2018	Death of 1 cattle on “pasture 2,” originally from “herd 2”
	Remaining herd moved to another pasture (“pasture 4”)
23rd June 2018	Death of 1 cattle, originally from “herd 2”, on “pasture 4”
25th June 2018	Death of 1 cattle, originally from “herd 2”, on “pasture 4”
3rd July 2018	Death of 1 cattle, originally from “herd 2”, on “pasture 4”
	Treatment of the entire remaining herd with 0.85 mg/kg imidocarb (Imizol®) i.m.
End of May 2019	One herd moved onto “pasture 2” and a few days later onto “pasture 5”
10th June 2019	Death of 3 cattle on “pasture 5”
	Treatment of the entire remaining herd with 0.85 mg/kg imidocarb (Imizol®) i.m.
10th September 2019	Death of 1 cow on “pasture 2,” several other cattle showed haemoglobinuria
	Treatment of the entire herd with 0.85 mg/kg imidocarb (Imizol®) i.m.

The dead bull as well as three of the cows were subjected to a pathological examination. All four animals were in good body condition, showed multifocal subcutaneous oedemas, mild jaundice, splenomegaly, haemoglobinuric nephrosis and dark brown urine. Infestation with adult and nymphal *I. ricinus* was also noted (19).

Despite moving the herd, further deaths occurred, including eight cows from a second herd, which had previously grazed on “pasture 3” (adjacent to “pasture 1”) and had joined the first herd to nurse the orphaned calves while on “pasture 2” (Table 1). On June 20th, two clinically affected cows were taken to the Clinic for Cattle, University of Veterinary Medicine Hannover. The two cows showed tick infestation, haemoglobinuria, reduced feed uptake, depression, and reluctance to move. On clinical examination, an elevated body temperature and increased heart rate (140/min, pounding beat) with a physiological respiratory rate (36 breaths/min) were noted. The rumen was hypo-motile and visible mucous membranes were pale and jaundiced (19).

### *Babesia* Detection in Cattle

All blood samples in the present study were taken for diagnostic purposes at the request of the animal owner. To confirm the suspected diagnosis “babesiosis,” blood smears from symptomatic animals (adult cows) were prepared from auricular venous blood, air dried, fixed with methanol and stained according to Giemsa (20). Blood smears were then examined microscopically at 1,000× magnification with oil, and parasitaemia was determined by evaluating 1,000 erythrocytes.

To confirm *B. divergens* infection and to screen further blood samples (taken from the jugular vein and stored at 8°C until processing), DNA was isolated with the NucleoSpin 8 Blood kit (Macherey-Nagel GmbH & Co. KG, Düren, Germany) according to the manufacturer's instructions, followed by a genus-specific PCR amplifying a 425 bp fragment of the 18S rRNA gene by use of primers BJ1 and BN2 (21). The 25 μl reaction volume contained 0.5 μl DreamTaq® Polymerase (Thermo Fisher Scientific Inc., Waltham, MA, USA), 2.5 μl 10 × Buffer, 0.5 μl of dNTPs (10 mM), 0.5 μl of each primer (10 μM each), 15.5 μl deionized water, and 5 μl template DNA. The following thermoprofile was used: initial denaturation at 95°C for 3 min, followed by 40 cycles denaturation at 94°C for 30 sec, annealing at 55°C for 30 s and extension at 72°C for 1 min, and final extension at 72°C for 10 min. After electrophoresis on 1.5% agarose gels stained with GelRed® (Biotium Inc., Fremont, CA, USA), the obtained amplicons were visualized under UV light and custom Sanger-sequenced at Microsynth Seqlab Sequencing Laboratories (Göttingen, Germany).

### Seroepidemiological Investigations

To assess patterns of *B. divergens* exposure with the aim of developing a grazing strategy limiting clinical disease, serological testing was carried out at two occasions: In July 2018 (after the first outbreak) on 25 animals of the initially affected herd (11 calves and 14 cows), and in March 2020 on 95 animals (4 calves, 30 heifers, and 61 cows) representing different herds. Serum was prepared from blood samples taken from the jugular vein by centrifugation at 2,300 × g for 15 min and stored at −20°C until analysis. Anti-*B. divergens* antibodies were measured in a commercial indirect immunofluorescence test (MegaFLUO® *Babesia divergens*, MEGACOR Diagnostik GmbH, Hoerbranz, Austria) according to the manufacturer's instructions.

### Investigation of *Babesia* Prevalence in Ticks

Questing ticks were collected on three different pastures of the farm, where clinical babesiosis had occurred (pastures 1, 2, and 5, Figure 1), by the flagging method in June, September and October 2018 as well as in March and June 2019. Ticks were microscopically identified based on morphological keys (22). DNA was extracted from whole individual adult and nymphal ticks, as well as pools consisting of 15 larvae each, by means of the NucleoSpin 8 Blood kit (Macherey-Nagel GmbH & Co. KG, Düren, Germany) as described in Tappe and Strube (23). To assess *Babesia* spp. prevalence in ticks, PCR and sequencing of the partial 18S rRNA gene were performed as described above. Obtained sequences were compared to published *Babesia* sequences using the NCBI Blast algorithm.

## Results

### Diagnosis and Treatment, Year 2018

**Figure 2:**
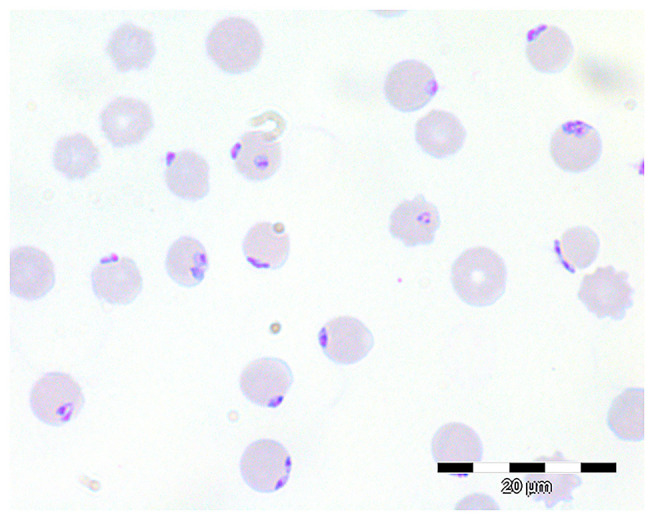
Giemsa-stained blood smear showing numerous intra-erythrocytic *B. divergens* stages.

In the blood smears of the two symptomatic animals treated in the Clinic for Cattle, typical small *Babesia divergens* merozoites, located at the periphery of erythrocytes, were detected (Figure 2). Parasitaemia amounted to 37.0 and 39.3%, respectively (19). Blood samples were also subjected to PCR and *Babesia divergens* infection was confirmed, as obtained partial 18S rRNA sequences showed 100% nucleotide identity to published *B. divergens* sequences (e.g., LC477143, 100% query cover). One of the animals recovered after treatment with imidocarb diproprionate (Carbesia®, MSD Animal Health, Luzern, Switzerland; 0.85 mg/kg body mass i. m.), whereas the other one died. On July 3rd 2018, the entire remaining herd was treated with imidocarb diproprionate (Imizol®, Intervet UK Ltd., Milton Keynes, UK; 0.85 mg/kg estimated body mass i. m.). Overall, the number of fatalities in 2018 due to babesiosis amounted to 21 animals (Table 1), all of these were over 2 years of age.

### Epidemiology in Cattle, Years 2019–2020

During winter 2018/2019, all animals were housed and new herds were formed in spring 2019. At the beginning of June 2019, all adult animals turned out onto “pasture 1” (cf. Figure 1) were treated prophylactically with imidocarb diproprionate (Imizol®, Intervet UK Ltd., Milton Keynes, UK; 0.85 mg/kg body mass i. m.). At the same time, another herd was moved onto “pasture 2” and a few days later onto “pasture 5” directly adjacent to “pasture 2.” On June 10th, three cows were found dead on this pasture, while others displayed clinical signs of babesiosis. These animals had not been part of the affected herd during the previous year's outbreak. *Babesia divergens* infection was confirmed by Giemsa-stained blood smears and PCR. In consequence, imidocarb treatment (Imizol®, Intervet UK Ltd., Milton Keynes, UK; 0.85 mg/kg body mass i. m.) of all animals on “pasture 5” was initiated. The herd was later moved onto “pasture 2” again. Despite treatment, on September 10th 2019, another cow was found dead in this herd after displaying haemoglobinuria. Babesiosis can be assumed as the likely cause of death, as an engorged female *I. ricinus* tick removed from the animal was tested positive for *B. divergens* by PCR.

In 2020, no clinical signs of babesiosis were noted to this date (May 13th, 2020).

### Seroepidemiology of the Different Cattle Herds, 2018–2020

On July 18th 2018, blood samples were taken from the entire remaining stock of the farm (five herds, 137 animals). *Babesia* DNA was detected in 5.8% of these samples (8/137) by 18S rRNA PCR, including one calf and seven cows from the clinically affected herd. In contrast, no *Babesia*-positive animals were detected in the other four herds.

In addition, sera from 25 animals (14 cows and 11 calves) of the affected herd were tested for the presence of anti-*B. divergens* antibodies by indirect immunofluorescence (MegaFLUO® *Babesia divergens*, Megacor Diagnostik GmbH, Hörbranz, Austria). Titres indicating pathogen exposure (≥1:64) were detected in all of the 14 adult cows and 2/11 calves (18.18%), while three further calves (27.27%) showed a borderline result (<1:64, ≥1:16) and six (54.55%) were seronegative (<1:16).

In March 2020, further serological testing was carried out, including 83 animals that had grazed in 2018, in 2019 or during both years on pastures where babesiosis cases occurred (“pasture 1” and “pasture 2”/“pasture 5,” cf. Figure 1), and 12 animals that had grazed on a different area of the farm. Among the latter 12 animals, 11 (91.67%) were seronegative, while one animal (8.33%) showed a borderline result.

**Figure 3:**
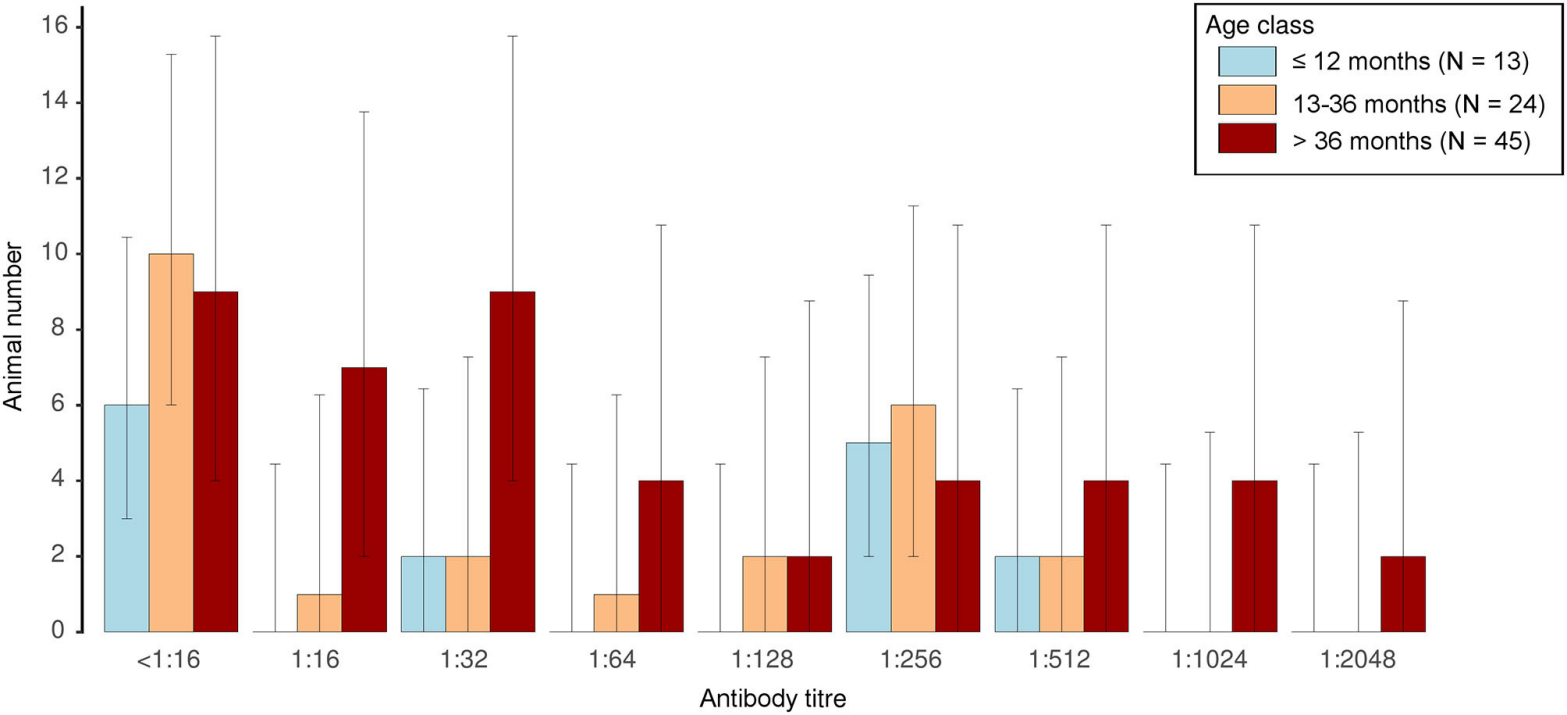
Distribution of anti-*B. divergens* antibody titres as determined by indirect immunofluorescence in animals of different age classes. Error bars depict 95% confidence intervals.

Among the 83 potentially exposed animals, positive titres were detected in 36 animals (43.37%), while 21 animals (25.30%) showed a borderline result and 26 (31.33%) were seronegative. No significant difference in the distribution of titres between different age categories [≤12 months [*n* = 13], 13–36 months [*n* = 24], >36 months [*n* = 45]] was found in this group (Kruskal-Wallis rank sum test, χ^2^ = 1.62, *P* = 0.444, Figure 3). Previous treatment with imidocarb diproprionate also did not seem to affect titres, as none of the animals in the youngest age group had been treated, whereas only 16.67% (4/24) of the animals aged 13–36 months, but 88.89% (40/45) of the animals >36 months had been treated during the last 2 years.

Thirteen animals were tested both in 2018 and 2020 (Table 2). Four of these animals (30.77%) showed positive titres in both years, while the titres of eight animals (61.54%) had declined from a positive result in 2018 to a negative or borderline result in 2020. One animal (7.69%) was seronegative in both years.

**Table 2:** Evolution of anti-*B. divergens* antibody titres in animals tested in 2018 and 2020.

**Animal**	**July 2018**		**March 2020**		
	Titre^*^	**Interpretation**	**Titre**	**Interpretation**	**Comment**
1	≥1:128	positive	<1:16	negative	
2	<1:16	negative	<1:16	negative	
3	≥1:128	positive	1:32	borderline	
4	≥1:128	positive	1:512	positive	Clinical babesiosis (2018)
5	≥1:128	positive	1:256	positive	
6	≥1:128	positive	1:32	borderline	
7	≥1:128	positive	1:32	borderline	
8	≥1:128	positive	1:64	positive	
9	≥1:128	positive	1:32	borderline	Clinical babesiosis (2018)
10	1:64	positive	1:32	borderline	
11	≥1:128	positive	1:32	borderline	Clinical babesiosis (2018)
12	≥1:128	positive	1:128	positive	
13	≥1:128	positive	1:16	borderline	

* *In 2018, sera were only tested up to a dilution of 1:128*.

### *Babesia* spp. Prevalence in the Tick Host

A total of 1,430 ticks were collected from June 2018 to June 2019 (227 adult ticks, 648 nymphs and 555 larvae), 1,152 of them on “pasture 1,” 245 on “pasture 2” and 33 on “pasture 5” (Figure 1, Table 3). All ticks were identified as *I. ricinus* based on morphological criteria.

**Table 3:** *Babesia* prevalence and *Babesia* species identification in ticks collected on a Northern German cattle farm affected by *Babesia divergens*.

		**Pasture 1**		**Pasture 2**		**Pasture 5**	
		**Positive/collected ticks (%)**	***Babesia* spp**.	**Positive/collected ticks (%)**	***Babesia* spp**.	**Positive/collected ticks (%)**	***Babesia* spp**.
June 2018	A	0/19 (0.00%)	-	0/10 (0.00%)	-	-	-
	N	1/184 (0.54%)	*B. microti*	1/76 (1.32%)	*B. capreoli*	-	-
	L	0/555 (0.00%)	-	-	-	-	-
September 2018	A	1/20 (5.00%)	*B. microti*	-	-	-	-
	N	0/66 (0.00%)	-	-	-	-	-
October 2018	A	1/30 (3.33%)	*B. microti*	-	-	-	-
	N	6/88 (6.82%)	4× *B. microti*, 2× *B. venatorum*	-	-	-	-
March 2019	A	1/130 (0.77%)	*B. venatorum*	-	-	-	-
	N	0/60 (0.00%)	-	-	-	-	-
June 2019	A	-	-	0/9 (0.00%)	-	0/9 (0.00%)	-
	N	-	-	3/150 (2.00%)	*B. venatorum*	0/24 (0.00%)	-
**Total**		**9/1152 (0.78%)**		**4/245 (1.63%)**		**0/33 (0.00%)**	

*A, adult ticks, N, nymphal ticks, L, tick larvae*.

Overall, *Babesia* prevalence in ticks amounted to 0.91% (13/1,430). DNA of *B. divergens* was not detected in any tick sample. Obtained *Babesia* sequences were 100% identical to published *B. microti, B. venatorum* or *B. capreoli* sequences, respectively (e.g., EF413181, MK641014, FJ944828; 100% query cover each). Obtained sequences were deposited in GenBank under the accession numbers MT657323-MT657326. DNA of *B. microti* was found in 0.49% (7/1,430) of ticks, namely in 0.77% (5/648) of nymphs and 0.88% (2/227) of adult ticks. Overall prevalence of *B. venatorum* was 0.42% (6/1,430), 0.77% (5/648) in nymphs and 0.44% (1/227) in adults, while *B. capreoli* was detected in one tick only (0.07%; 0.15% [1/648] in nymphs, 0.00% [0/277] in adults). All of the tested 37 larvae pools were *Babesia*-negative. Detailed results are presented in Table 3.

## Discussion

Bovine babesiosis due to *B. divergens* has been declining in many parts of Europe during the past decades (12–14). This decline has probably lead to a loss of herd immunity resulting in an unstable epidemiologic situation and a decrease in awareness for the disease among veterinarians and farmers (12). At the same time, there is a permanent risk of *B. divergens* (re-)introduction by movement of wildlife, migratory birds or domestic animals carrying infected ticks, or by movement of premune, subclinically infected cattle. L'Hostis and Seegers (17) identified several risk factors for the re-emergence of bovine babesiosis in Europe, including changing management practices, e.g., a return to more extensive farming systems, longer grazing periods and a decrease in the use of antiparasitic chemotherapy, as well as an increase in tick numbers due to changes in landscape, climate, and availability of wildlife hosts.

In the present case, a babesiosis outbreak occurred on a farm without any previous history of bovine babesiosis. It is likely that *B. divergens* was introduced onto the farm's pastures via transport of infected ticks by migratory birds, as the area serves as a bird breeding and overwintering area and migration stop. Another possibility is transport of infected ticks by dogs, which had previously traveled to endemic regions, being walked near the pastures. The affected pastures are located directly adjacent to a forest, and are interspersed with pockets of forest and hedges, which provide suitable habitats for a large number of ticks. Moreover, wild cervids serving as hosts for adult and nymphal *I. ricinus*, e.g., roe deer, were probably abundant, as cervid-associated *Babesia*, namely *B. capreoli* and *B. venatorum*, were detected in the collected ticks. Additionally, detection of *B. microti* indicates abundance of rodents, which are preferred hosts of *I. ricinus* larvae. Like *B. divergens, B. microti* as well as *B. venatorum* are of zoonotic importance (24), although clinical *B. microti* infections of humans have so far only been acquired in the Americas (3), suggesting that European *B. microti* strains may be less pathogenic. Here, *B. microti* and *B. venatorum* were detected at similar prevalences in adult and nymphal ticks (0.88%/0.77% and 0.44%/0.77%, respectively) compared to studies from other European countries (25–27).

In contrast, despite collection and testing of 1,430 ticks, including all life stages, from the pastures where clinical babesiosis cases occurred, no *B. divergens*-positive ticks were detected. Similarly, Lempereur et al. (28) found no *B. divergens*-positive ticks among 805 specimens collected on eight Belgian farms with a known history of bovine babesiosis. Possibly, *B. divergens*-infected ticks occurred only in small foci on the pastures, which were missed during flagging. Another reason may be that the main activity peak of infected ticks was missed, although flagging was conducted during several different seasons.

Despite the fact that no *B. divergens*-positive ticks were found, the epidemiological data suggest a geographical spread of infected ticks on the farm, as the clinical cases in 2019 occurred on the pasture where the affected herd had been moved to after the first outbreak the year before. As the animals were not treated with an acaricide before movement, they may have carried infected ticks, while also serving as a source of infection for uninfected ticks on the second pasture.

The first outbreak in 2018 was characterized by a delay in diagnosis and difficulties to obtain sufficient quantities of imidocarb, which is not licensed and thus not marketed in Germany, and thus a high case fatality rate. In consequence, the economic loss for the affected farm was considerable. Similarly, Zintl et al. (12) reported an increased case fatality rate of bovine babesiosis in Ireland and attributed this to a lack of awareness for the disease among veterinarians and farmers. In the present case, poisoning was initially suspected before the final diagnosis was made almost 2 weeks after the first fatalities. Here, it should be noted that the *post mortem* diagnosis of bovine babesiosis is challenging (23). The method of choice for diagnosing acute *B. divergens* infection is inspection of stained blood smears (29). Thorough daily inspection of all individual cattle in a herd by farmers, especially in extensively managed systems, is also important to detect clinical signs like haemoglobinuria as early as possible, as these may only be present in a few animals at the beginning of an outbreak.

Regarding treatment of bovine babesiosis, only imidocarb diproprionate is available on the European market, but it is not licensed in all European countries. The substance is characterized by a long half-life and slow elimination (30), and therefore also exerts a prophylactic effect. At 2.5 times the therapeutic dose (2.125 mg/kg body mass), this prophylactic effect lasts for up to 4 weeks according to the manufacturer (31). Here, prophylactic treatment at the therapeutic dose was attempted, and may have contributed to the reduced number of clinical cases in 2019 as compared to 2018, in addition to a build-up of natural immunity in previously exposed cattle, which seems to be independent of imidocarb treatment as indicated by the serological results of this study. Furthermore, treatment of symptomatic animals occurred earlier in 2019, reducing the fatality rate.

A disadvantage of imidocarb is the long withdrawal time on meat of 213 days, which may be problematic for many farmers. Alternatively, acaricides such as organophosphates, pyrethroids or carbamates are licensed against tick infestation in cattle in some countries. However, to protect cattle during the entire tick season, treatment needs to be repeated regularly. For example, a flumethrin pour-on formulation, the only acaricide licensed against tick infestation of cattle in Germany, needs to be applied every 3 weeks. Thus, chemoprophylaxis of bovine babesiosis may not be practicable on all farms, especially in extensively managed systems. Live attenuated vaccines have been used with variable success in the past, but are currently not commercially available (32). Further options for affected farms are to discontinue using pastures with known transmission foci for several years or to manage herds in an attempt to create enzootic stability by ensuring that calves gain sufficient immunity through natural exposure in their first year of life (33). Here, antibody titres were assessed in the majority of stock before the second pasture season following the initial outbreak, in order to develop a grazing strategy limiting clinical disease. As most babesiosis cases occur in seronegative animals (9), these animals can be chosen for prophylactic treatment or grazing on non-affected pastures, if they are older than 9 months. However, titres vary from year to year as they may decline during the housing period (34), which was also evident in the 13 retested animals in this case, and large scale serological testing of entire herds before each grazing season may not be an economical long-term solution.

In conclusion, although disease incidence is rather low, bovine babesiosis remains a disease of veterinary importance in Europe, which may cause considerable economic losses and may be challenging to manage when (re-)emerging in non-endemic areas. Furthermore, *B. divergens* is of zoonotic concern. In addition to *B. divergens*, further *Babesia* spp. of zoonotic importance circulate in European tick populations. In the present study, *B. microti* and *B. venatorum* were detected in a popular recreational area, indicating a potential risk for humans.

## Data Availability Statement

Obtained sequences were deposited in GenBank under the accession numbers MT657323-MT657326.

## Ethics Statement

Ethical review and approval was not required for the animal study because all samples from animals in the present study were taken for diagnostic purposes for the benefit of the animals and at the request of the animal owner and thus did not require ethical approval. Written informed consent was obtained from the owners for the participation of their animals in this study.

## Author Contributions

AS, FL, SE, MHöl, KH, AL, CG, and DB performed laboratory work. CS designed and MHöl, JR, MHoe, PW, WB, and CS coordinated the study. AS drafted the manuscript. All authors participated in data analysis and interpretation, reviewed the manuscript draft, read, and approved the final manuscript.

## Conflict of Interest

DB is an employee of Veterinary Laboratory Freiburg GmbH. CG is an employee of MEGACOR Diagnostik GmbH. Study data collection and interpretation is completely independent from the companies' opinion and DB and CG declare that there is no conflict with commercial interests. The remaining authors declare that the research was conducted in the absence of any commercial or financial relationships that could be construed as a potential conflict of interest.
